# For Whom the Clock Ticks: Clinical Chronobiology for Infectious Diseases

**DOI:** 10.3389/fimmu.2020.01457

**Published:** 2020-07-09

**Authors:** Aïssatou Bailo Diallo, Benjamin Coiffard, Marc Leone, Soraya Mezouar, Jean-Louis Mege

**Affiliations:** ^1^Aix-Marseille Univ, MEPHI, IRD, AP-HM, Marseille, France; ^2^IHU-Méditerranée Infection, Marseille, France; ^3^Aix-Marseille Univ, AP-HM, Hôpital Nord, Médecine Intensive-Réanimation, Marseille, France; ^4^Aix-Marseille Univ, AP-HM, CHU Hôpital Nord, Service d'Anesthésie et de Réanimation, Marseille, France; ^5^AP-HM, UF Immunologie, Marseille, France

**Keywords:** circadian rhythm, clock genes, microorganisms, microbiota, immune response, infectious diseases

## Abstract

The host defense against pathogens varies among individuals. Among the factors influencing host response, those associated with circadian disruptions are emerging. These latter depend on molecular clocks, which control the two partners of host defense: microbes and immune system. There is some evidence that infections are closely related to circadian rhythms in terms of susceptibility, clinical presentation and severity. In this review, we overview what is known about circadian rhythms in infectious diseases and update the knowledge about circadian rhythms in immune system, pathogens and vectors. This heuristic approach opens a new fascinating field of time-based personalized treatment of infected patients.

## Introduction

The defense against pathogens varies among individuals. Several heritable and non-heritable influences may account for these inter-individual variations. It now appears that the most important variations of host response among individuals include those associated with circadian disruptions (1). The circadian rhythm (Latin origin: *circa*: almost; *dies*: day), also called biological clock, is found in all living organisms, including eukaryotes and prokaryotes, and is defined as a period of about 24 h, temperature-compensated and entrained by a Zeitgeber (German name for synchronizer) (2, 3). The idea that an intrinsic rhythmicity governs host adaptation to the environment was introduced three centuries ago with the movement of mimosa leaves and, one century later, by the demonstration of gene-encoded rhythm in *Drosophila melanogaster* (4–7). This fascinating field of research is expanding with the recent awarding of the Nobel Prize to Hall, Rosbash and Young (8).

The function of circadian rhythms is to anticipate the changes and cycles of the surrounding world, such as day/night cycle and earth rotation, in order to optimize the response of organisms to these changes (9, 10). In mammals, including humans and non-human primates, this biological rhythmicity is coordinated by a central molecular oscillator, the suprachiasmatic nucleus (SCN), which ensures synchronization with light/dark cycle through specialized neurons from retina that receive photonic signals and the SCN then sends projections to other regions of the brain with local clocks such as immune organs, leading to activation of peripheral clocks (11–14). Interestingly, the SCN triggers a circadian rhythm independently of any temporal reference and is an autonomous timekeeper (15). In peripheral tissues, most cells have an internal molecular clock (16), but the function of these specific oscillators is not fully understood.

**Figure 1:**
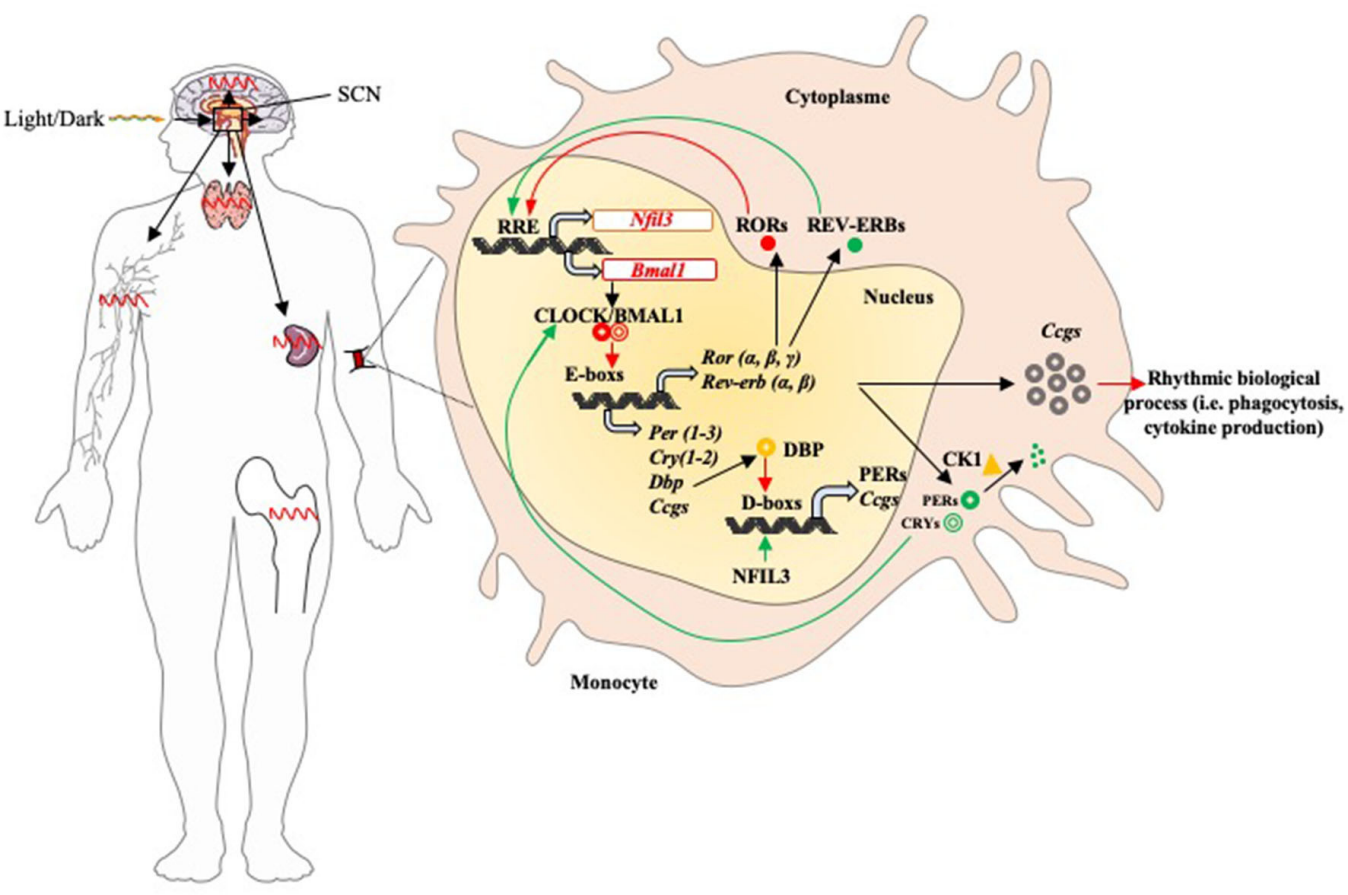
Circadian rhythm of the immune system components. The suprachiasmatic nucleus is the central oscillator that mediates all circadian variations in humans. It receives synchronization information with the day/night cycle *via* its retinal connection and then sends projections to other regions of the brain with local clocks such as immune organs (lymph nodes, spleen, thymus, and bone marrow) and activates peripheral clocks (activation of circadian feedback loops). In most immune cells (represented by functions such as phagocytosis and cytokine production), the feedback loops function as follows: once its gene is expressed, the BMAL1 protein dimerizes with CLOCK in the nucleus; the CLOCK/BMAL1 heterodimer binds to the E-box promoter sequences and induces expression of *Pers, Crys, Reverbs, Rors*, and *Ccgs*. The PERs and CRYs proteins dimerize in the presence of the casein kinase 1 (Ck1), then return to the nucleus to prevent the binding of the dimer CLOCK/BMAL1 on DNA. PERs proteins are phosphorylated by Ck1 and then degraded by the proteasome. *REVERBS* and *RORs* have antagonistic effects on *Bmal1* expression: they bind the promoter of its gene and then induce (RORs) or inhibit (REVERBs) its expression. DBP protein induces the expression of *Pers* and *Ccgs* by binding D-box promoter sequences; it is inhibited by NFIL3. Positive and negative factors are represented by red and green arrows, respectively.

The molecular mechanisms of circadian rhythms have been well-investigated so far. It involves the clock genes present in two main feedback loops (Figure 1). First, the positive loop consists of the genes encoding circadian locomotor output cycles kaput (CLOCK), brain and muscle arnt-like protein 1 (BMAL1), and retinoic acid-related orphan receptor α, β and γ (RORs) proteins. Second, the negative loop involves the genes encoding Period (PER) 1, 2, 3, cryptochrome (CRY) 1, 2 and REVERB-α, -β (also called NR1D1/2 or nuclear receptor subfamily 1 group D) proteins (17, 18). In most somatic cells, the transcription factor formed by the dimerization of BMAL1 and CLOCK proteins binds the E-box sequences of the promoters of the other clock genes (*Per, Cry, Reverb*, and *ROR*), inducing their expression and translation (19). The CLOCK/BMAL1 transcription factor controls a third circadian regulatory loop, DBP (D-box binding protein) by binding the D-box sequences of gene promoters (9). Following the action of the dimer CLOCK/BMAL1, the clock-controlled genes (*Ccgs*) are expressed. These latter control 30% of the mammalian genome and regulate numerous physiological functions (16), including body temperature, blood pressure, hormone concentrations, blood circulation, urine output, metabolism, hair growth and immune system (14). In this context, during pathological conditions the involvement of circadian rhythm is now admitted but its investigation remains to date complex (20).

Here, we will summarize what is known about rhythmicity in infectious diseases. Then, we will update the knowledge about circadian rhythms in immune system, pathogens and vectors. Finally, we will translate this heuristic approach into a fascinating process for time-based personalized treatments of infected patients.

## Circadian Rhythms of Immune Effectors, Pathogens and Vectors in Infections

The occurrence of infectious diseases results from the conjunction of different factors including the ability of the host to coordinate the immune response, the nature and the virulence of the microorganisms and, sometimes, the presence of vectors such as mosquitoes. If the rhythms of immune system have been a source of recent reviews, those of pathogens and vectors are less investigated. We will summarize what is known about circadian rhythms of the immune system, microbes and vectors with a special attention to microbiota.

### Circadian Rhythms of Immune Effectors

It is well-established that the immune response varies according to circadian rhythms. These variations concern innate and adaptive immune responses at both quantitative and qualitative levels. At the quantitative level, the circulating number of hematopoietic stem and progenitor cells, and most mature leukocytes increases during the resting phase for rodents and the night for humans (21). The migration of immune cells to the tissues, a major phase of anti-microbial response, occurs preferentially during the active phase. This response also involves neuro-mediators released locally by sympathetic nerves, thus underlying the role of central pacemaker in addition to peripheral clocks (22). It has recently been shown that neutrophil traffic is regulated through a timer program. The deletion of aryl hydrocarbon receptor nuclear translocator-like protein 1 (*Arntl*) and *Cxcr2* genes prevents diurnal rhythms, called neutrophil aging (23). The homing of circulating lymphocytes into lymph nodes and their egress into efferent lymphatic vessels obey to a rhythmic process. The egress of cells is based on circadian variations of CCR7 production and that of its ligand CCL21 in both T and B lymphocytes (24).

The immune functions are modulated by circadian control. Regarding the innate immune system, functions such as particle uptake and release of oxygen derivatives and cytokines exhibit circadian rhythms (25, 26). Regarding the adaptive immunity, there is evidence that the functions of T and B lymphocytes also exhibit circadian rhythms. Indeed, the production of antibodies in response to thymo-dependent and thymo-independent antigens oscillates with melatonin rhythm in mice; melatonin suppression is associated with increased levels of specific antibodies, which are corrected by the addition of melatonin and light (27). Similarly, immunoglobulin (Ig)-E levels and IgE-mediated allergic responses are regulated by molecular clock in mice (28) and human mast cells; similar results are obtained in human eosinophils activated with N-formyl-methionly-leucyl-phenylalanine, a canonical chemotactic peptide (29).

In mice, the peak of salivary IgA levels occurs during the night; this response seems to be under the control of a central pacemaker (30). The lessons from mice invalidated for clock genes underline the role of these genes in the ontogeny of B lymphocytes and plasma cells (31) The functions of T lymphocytes are also controlled in a circadian manner. The proliferative response of T lymphocytes to mitogens is strongly rhythmic and is impaired when clock genes are mutated (32). In addition, the clock genes are involved in the differentiation of Th17 cells. The Th17 cell development is suppressed by nuclear factor, interleulin-3 regulated (NFIL3), a circadian-regulated transcription factor; *Nfil3* and related orphan receptor gamma (*Ror*γ*t*) are expressed in CD4^+^ T cells rhythmically during dark phase and light phase, respectively (33, 34). The development of regulatory T cells producing interleukin (IL)-10 is controlled by melatonin, suggesting that all coordination of the immune response is under circadian control (34).

These results clearly show that the immune system is under the control of peripheral clocks. It is also regulated by hormones and neuro-mediators that reflect the activity of central pacemaker. The hypothalamic-pituitary-adrenal axis is activated in response to stress and appears synchronized to glucocorticoid circadian rhythms. Hence, oscillations of the lymphocyte number in humans are inversely correlated with diurnal rhythm of glucocorticoid production (35). It is likely that the rhythmicity of the immune response involves numerous mechanisms, including the contribution of light variation.

### Circadian Rhythms in Microbes and Pathogens

For a long time, it was believed that circadian rhythms are not expressed by single-cell organisms such as prokaryotes. One reason for this belief was the idea that organisms duplicating more than once a day do not need a rhythm longer than their life cycle (36). In the 1980's, circadian rhythm was found present in cyanobacteria (37–39), photoautotrophic organisms that produce oxygen by photosynthesis as plants and eukaryotic algae (40, 41). In cyanobacteria, photosynthesis occurs in daylight, whereas nitrogen production peaks during the night (42). It has also been shown a circadian variation of the expression of most genes implicated in cell division, chromatin compaction and photosynthesis (43).

**Figure 2:**
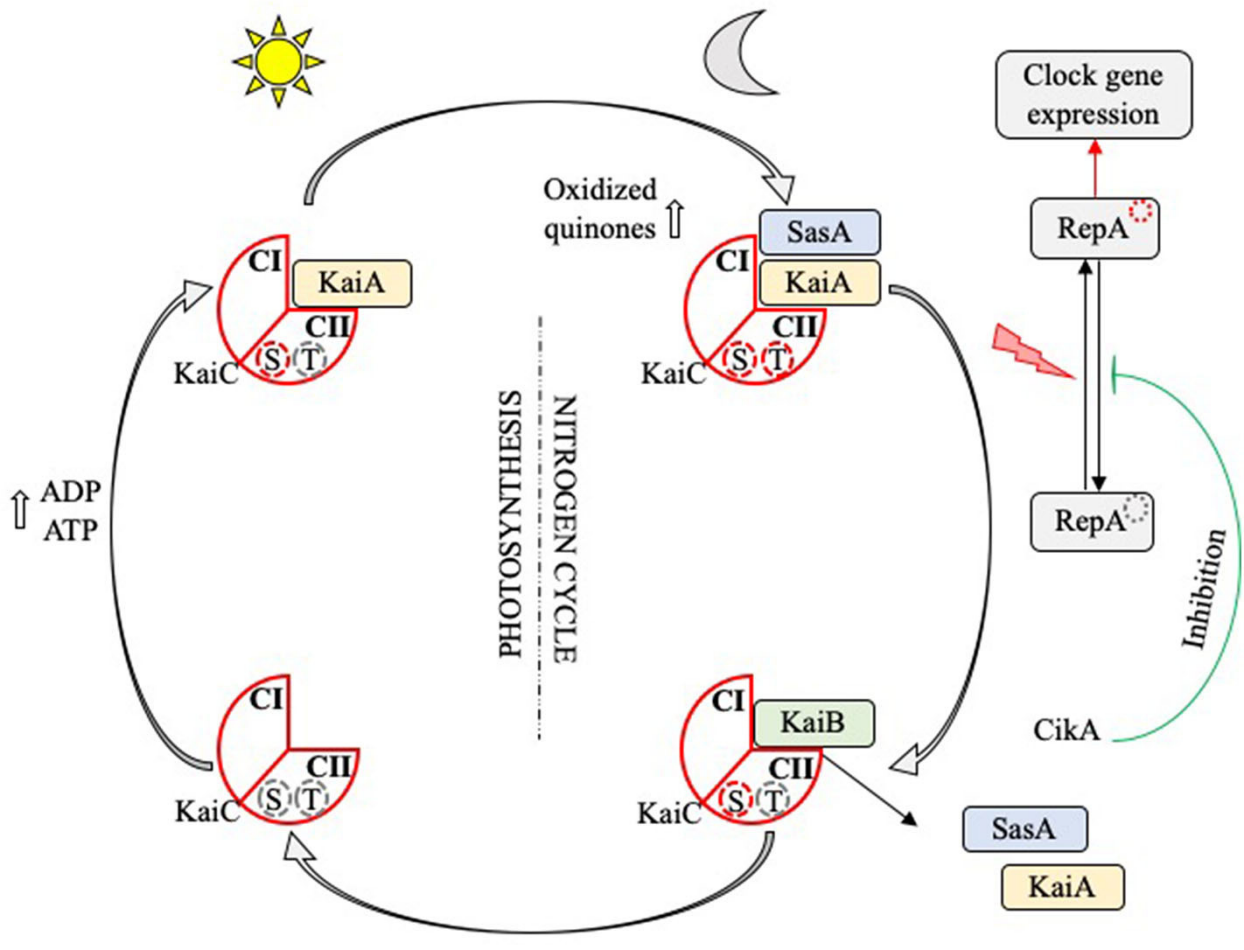
Circadian rhythm in cyanobacteria. The KaiC protein (red diamond) has two loop domains (CI and CII) and serine (S) and threonine (T) phosphorylation sites. At dawn, the increase in the ADP/ATP ratio, which reaches its maximum at noon, leads to the fixation of *KaiA* (yellow rectangle) on the CII domain, the phosphorylation of the T site and the induction of a training signal of photosynthesis elements. At nightfall, the increase in oxidized quinones following the initiation of the nitrogen cycle leads to the phosphorylation of the S site; *KaiC* is doubly phosphorylated. *SasA* (blue rectangle) is then fixed on the CI domain and activates the transcription factor *RepA*, which in turn induces expression of the target genes involved in the clock loop or others functions such as their multiplication. Finally, *KaiB* (green rectangle) releases *KaiA* and *SasA*, disable *RepA via* its interaction with the repressor *CikA*, and end up dephosphorylating *KaiC* which first finds itself serine phosphorylated.

Mechanistic studies have enabled the identification of clock genes responsible for circadian rhythms have been recently discovered in most organisms including fungi, algae and photosynthetic bacteria; they are named *Frq, Ibp*, and *Kai*, respectively (44, 45). Regarding cyanobacteria, the central biochemical oscillator of their circadian rhythm is represented by three proteins encoded by the *KaiABC* cluster (10, 46, 47), SasA, CikA, and RepA, which control the timing training, and output of the cyanobacterial cell signaling process (48). Several studies have examined how functions the circadian loop of cyanobacteria (40, 42, 49–51). A theoretical model of the *KaiABC* oscillator suggests that ATP hydrolysis is a driving mechanism of phosphorylation oscillations and that the frequency of ATP hydrolysis in individual *KaiC* molecules is correlated with the circadian rhythm frequency (50) (Figure 2). The reconstitution of cyanobacterium circadian loop *in vitro* is associated with oscillations of Kai proteins (52, 53), validating the theoretical approach of the *KaiABC* oscillator.

The belief that circadian rhythms are specific of cyanobacteria and that eubacteria are “non-circadian” should be revisited. Using a bioinformatics approach, homologs of *KaiB* and *KaiC* genes have been found in non-circadian bacteria including *Pseudomonas* species and in archaea (46). The functions of non-cyanobacterial Kai proteins have not been described, and it remains unknown whether these proteins interact with input and output factors (54, 55). However, the *KaiABC* oscillator, when expressed into the “non-circadian” bacterium *Escherichia coli*, remains functional (56), suggesting that “non-circadian” bacteria possess the armaturium needed to circadian rhythms. We recently identified the *RadA* gene from *E. coli* as a *KaiC* structural homolog using a bioinformatic approach and showed the persistence of circadian rhythm in *RadA*^−/−^ and *RecA*^−/−^ mutants, suggesting that *RadA* is not the generator of circadian rhythm of *E. coli*. Hence, it does not play the same role than *KaiC* in cyanobacteria (unpublished data). To date, the identification of circadian rhythms in eubacteria remains challenging for the scientific community and opens new insights in terms of antibiotic resistance that could provide an interesting new therapeutic approach.

The circadian rhythms have been also described in parasites *Plasmodium sp*. (57). With a blood stage lasting 24 h or multiple of 24 h, its rhythm was found to be associated with recurrent fever. It has been reported that circadian variations of parasites are influenced by variations of glycemia from high levels during the night to low levels during the day (58, 59). Therefore, the links between rhythmicity of parasites and host homeostasis have been suggested. Similarly, the appearance of filarial parasites is also rhythmic (60). The parasites have their own circadian rhythm, as exemplified by *Trypanosoma brucei* that can generate circadian expression in the absence of host cells (61).

### Circadian Rhythms in Vectors

The insect vectors have their own biological rhythm (62) that depends on the response to daily light/dark cycle and molecular clock activity. *Anopheles* mosquitoes that transmit malaria are active at dusk, so their bites occur at night during the host's resting phase. In contrast, *Aedes* mosquitoes that transmit dengue/yellow fever are day biters (63). The pathogens may affect vector circadian rhythms. The dengue virus is responsible for high amplitude of rhythmic locomotor activity of mosquitoes. *Aedes aegypti* exhibits diurnal rhythms that contribute to circadian transmission of Zika virus (64). The proteobacterium *Wolbachia*, an endosymbiont of arthropods, was reported to influence sleep time in flies (65). Recently, it has been shown that the daily activity of fly is affected by peripheral clocks and *Wolbachia* presence in flies (66).

The existence of biological rhythms in vectors may affect their resistance to toxic compounds. In *Anopheles gambiae*, the genes encoding the metabolic resistance to dichloro-diphenyl-trichloroethane (DDT) insecticide are rhythmically expressed (67, 68). Correlations between circadian enzymatic detoxification of insecticide and feeding time have been established (69). Thus, it appears that interactions between the circadian rhythms of the three partners involved in malaria transmission, namely mosquitoes, parasites and human host, lead to the development of the disease.

### Rhythms of Intestinal Microbiota and Medical Consequences

At the intersection of microbial world, immune response and homeostasis, the intestinal microbiota exhibit variations of circadian rhythms (70, 71). The human gastrointestinal tract contains a large number of microorganisms, of which the most studied are the bacteria (72). They contribute to maintain various host physiological functions such as immune defense and/or tolerance (73). Recently, the circadian rhythm of the intestinal microbiome composition as well as the functions of this microbial community related to the circadian rhythm have been reviewed (74). Although intestinal bacteria are not exposed to the light/dark cycle, they are subjected to circadian changes associated to host activities, including food intake and exposure to antibiotics (74, 75). Nevertheless, light variations affect the abundance of some microbes such as *Ruminococcus torques* known to affect gut barrier integrity (76) and that of *Clostridia* sp. (77). Interestingly, it has been reported that *Enterobacter aerogenes*, a gram-negative bacterium, is sensitive to melatonin known to be a circadian rhythm synchronizer and expresses circadian swarming and motility (78). These findings strengthen the hypothesis of circadian rhythms in prokaryotes.

The use of mouse models in which the intestinal microbiota is modulated or mice with a clock gene deficiency underlines the association between the microbiota and circadian rhythm. Liang et al. (79) found that the absolute number of fecal bacteria in C57BL/6 mice follows a circadian rhythm and that mice deficient in *Bmal1* gene show an altered daytime rhythm of microbiota in a sex-dependent manner. Mice deficient in *Per1/2* and disrupted sleep cycle show an almost total loss of rhythmic fluctuations of microbiota and present intestinal dysbiosis (80). The circadian rhythms of microbiota over 24-h periods also depend on the host. Hence, this rhythmicity disappears *in vitro* when bacteria are cultivated, suggesting a regulation by the host (81). Leone et al. (82) showed that the relationship between microbiota and circadian rhythms is affected by the type of diet in germ-free mice. Indeed, bacteria from the family *Lachnospiraceae* exhibit circadian rhythms under regular low-fat chow, which are absent in mice with high fat diet. The diabetic mice exhibit a loss of diurnal rhythms of bacteria such as *Akkermansia genus* (83).

The association between circadian variations of microbiota and mechanisms of nutrition-associated disorders has been recently reported. Based on the observation that circadian misalignment promotes the occurrence of obesity, it has been shown that disruption of circadian rhythms impacts gut microbial composition and risk of obesity in rodents. In the absence of microbiota, the recruitment of histone deacetylase 3 is defective and cyclical histone acetylation is lost, suggesting a relationship between microbiota rhythms and epigenetic changes (84, 85). In addition, a new target of diurnal rhythms of microbiota, the group 3 innate lymphoid cells (ILC3), has been recently reported (86). Indeed, a dysregulation of brain rhythmicity affects circadian rhythms of ILC3 and microbiota, suggesting that environmental light directly regulates diurnal variations of enteric ILC3. In summary, a disruption of the circadian rhythm, by mutation of clock genes or disruption of the wake/sleep cycle, leads to changes in the composition of the intestinal microbiota and metabolic changes in host, opening a new fascinating field of investigations.

## Circadian Rhythm in Infectious Diseases: From Experimental Studies to Clinics

The study of circadian rhythms in infectious diseases is more complex than the investigation of rhythmicity in immune effectors and microbes. It requires an integrated approach associating experimental investigations and clinical observations. The aim of this section is to discuss the impact of infection to host circadian rhythms, the role of circadian rhythms in the susceptibility and/or resistance of individuals to infection and finally the situation of septic syndromes.

### Impact of Infection on Host Circadian Rhythm

The evidence of circadian variations in infectious diseases due to bacteria, parasites and virus is based on clinical observations and on the use of animal models including mice invalidated for clock genes (Table 1).

**Table 1:** Circadian rhythm in infectious diseases.

**Pathogens**	**Circadian effects**	**Clock gene/protein**	**References**
*Streptococcus pneumoniae*	• The severity of the response depends on the time of the infection (higher at the beginning of the resting phase compared to the active phase) • Circadian phagocytosis in infected macrophages	Bmal1	(87, 88)
*Helicobacter pylori*	• Molecular clock disruption which is related to an increase of the infection	Bmal1	(89, 90)
*Listeria monocytogenes*	• Disruption of the circadian circulation of inflammatory monocytes to tissues	Bmal1	(91)
*Mycobacterium tuberculosis*	• Infected patients coughing is more common during the day	–	(92)
*Salmonella enterica* serovar Typhimurium	• Bacterial colonization of the colon is more pronounced during the resting phase than during the active phase	Clock	(93)
Hepatitis C Virus	• Patients with chronic HCV infection develop a disrupted circadian rhythm	Per2 REV-ERBα	(94–96)
Influenza A virus	• Mice infected before the onset of active phase exhibit higher mortality and morbidity than mice infected before the rest phase	Bmal1	(96)
Herpes simplex virus	• Viral replication 10-fold higher in mice infected during the resting phase vs. the active phase	–	(97)
*Trypanosoma brucei*	• Circadian disruption of body temperature and locomotive activity • Infected mice have a period of <24 h and abnormal activity during the resting phase.	Bmal1	(98)
*Trichuris muris*	• Loss of cyclic antigen presentation • The parasite persists in infected mice at night	Bmal1	(99)
*Plasmodium chabaudi*	• Alteration of the circadian rhythm of blood glucose (hypoglycemia at the end of the active phase)	Bmal1	(58)

During infection, central and peripheral circadian rhythms of the host may be altered. In infected mice, the interaction of *Streptococcus pneumonia* with epithelial cells account for circadian variations of pulmonary inflammation including the release of inflammatory mediators and recruitment of inflammatory cells. In the absence of *Bmal1* gene, the recruitment of neutrophils is increased in relation with a disruption of CXCL5-glucocorticoid receptor interaction (87). Moreover, the infection with *Helicobacter pylori*, that is responsible for gastritis and paves the way of gastric cancer, involves alterations in gastric acid secretion, which is regulated by circadian rhythm (89). *H. pylori* dysregulates the molecular clock in gastritis through the upregulation of *Bmal1* gene expression in gastric epithelial cells. Importantly, the expression of the *Bmal1* gene is also upregulated in gastric tissues from patients with atrophic gastritis and dramatically increased in patients with precancerous lesions, thus establishing a relationship between disrupted circadian rhythm and the severity of the infection. In addition, *H. pylori* disrupts the circadian rhythm of an important *Bmal1* target, the gene encoding tumor necrosis factor (*Tnf*), exacerbating the TNF production (90).

Disruption of circadian rhythm was also observed during intracellular bacterial infection. *Listeria monocytogenes* is a foodborne gram-positive bacillus known to be pathogen when T-cell mediated responses are impaired. The number of inflammatory monocytes considered as the effectors of inflammation and anti-*Listeria* immunity vary according to a circadian rhythm. Their circadian traffic to the tissues is disrupted by the myeloid impairment of *Bmal1* gene (91). Our team previously showed expression variations of clock genes in Q fever, a zoonosis due to *Coxiella burnetii*, an obligate intracellular bacterium. The analysis of microarrays performed to understand sexual dimorphism in *C. burnetii* infection shows that circadian genes (*Bmal1, Clock*, and *Per2*) are upregulated in the liver from infected female mice, as compared with healthy females and infected males (100). In patients, the *Per2* gene is more expressed in males with acute Q fever than in healthy volunteers (101), suggesting that the unexpected relationship between circadian rhythm and gender dimorphism of Q fever may be extended to other human infectious diseases.

Interestingly, it was reported that parasites seem to be able to synchronize circadian rhythm and immune response. Indeed, in mice infected by *Plasmodium chabaudi*, the frequency of parasite replication is related to the circadian rhythm of TNF expression (59). Indeed, increased TNF is associated with a higher frequency of non-replicating cyclic forms of trophozoites. Infection alters circadian rhythms of blood glucose with a hypoglycemia at the end of the active phase and a direct impact on parasites. The oscillations of TNF and glucose levels seemed critical for parasite replication (58). It is established that malaria due to *Plasmodium* sp. exhibit a periodicity of fever that is secondary to the burst of *Plasmodium*-infected red blood cells every 24–72 h (58). Mice infected with *T. brucei* have a period shorter than 24 h and abnormal activity during the resting phase. This model reproduces sleeping sickness considered a circadian disorder in which infected individuals experience somnolence during the day and insomnia during the night (102).

Different viruses alter circadian-regulated biological processes such as CD4^+^ T cell numeration in human immunodeficiency virus infection (103). Other interferences have been described for simian immunodeficiency virus, coxsackievirus A16 (hand, foot and mouth disease) and human T-lymphotropic virus (96). Liver circadian-regulated genes that are likely targets for hepatitis C virus (HCV) may affect viral hepatitis. Patients with chronic HCV infection develop a disrupted circadian rhythm characterized by altered sleep patterns, although the association of sleep disorders and reduced survival in patients is debated (94). HCV infection also leads to decreased expression of *Per2* and *Cry2* genes. The *Per2* gene is critical for the anti-viral response since its overexpression in a hepatocyte cell line reduces HCV replication and increases the expression of interferon-stimulated genes (95). On the other hand, liver-associated microRNA (miR)-122 is involved in HCV replication; miR122 is negatively regulated by *Rev-erb*α, strengthening the idea that HCV replication depends on the daily rhythm (104). The association between hepatitis and circadian rhythms has potential implications for the patient management. Hence, viral RNA levels rebound more frequently in patients infected with HCV and undergoing liver transplantation in the morning than in those transplanted in the afternoon (105). Hepatic circadian gene oscillation is associated with circadian rhythm and sleep in HCV infection (94).

Patients infected with respiratory viruses exhibit daily rhythms of clinical symptoms, such as increased nasal secretion and body temperature in the morning compared to the late evening in patients with cold or flu (106). Mice with deficient molecular clock are highly susceptible to virus infection, as shown by increased replication of influenza, respiratory syncytial and parainfluenza type 3 viruses. Hence, *Bmal1* appears as a major clock gene controlling viral replication (107). The *Bmal1* gene is also involved in the coordination of lung immune response to virus since the deletion of *Bmal1* gene exacerbates acute bronchiolitis secondary to *Sendai* virus or *influenza A* virus (96). In the same extend, the severity of influenza A infection is directly controlled by clock genes expressed in lung epithelium and natural killer (NK)1.1^+^ cells, a subset of NK cells (108). The alteration of host circadian rhythm was also reported for *Herpes simplex* virus (HSV). The pathogenicity of HSV results from the interplay of viruses with circadian rhythms. The replication of HSV depends on histone deacetylation, and transcriptional machinery of HSV is associated with histone acetyltransferase, a clock-controlled gene (109).

### Circadian Rhythms in Susceptibility and/or Resistance to Infection

Several studies reported that the susceptibility and the resistance to infections are associated with host circadian rhythm. More than 40 years ago, it was reported that susceptibility to *S. pneumoniae* is altered in blind and adrenalectomized mice with altered circadian rhythms (110). Recently, Kitchen et al. (88) reported that macrophages from *S. pneumonia* infected *Bmal1*-deficient-mice presented increase bacterial ingestion and cytoskeletal change both associated with an impaired function of RhoA pathway. The infection is more severe when mice are infected at the beginning of the resting phase than during the active phase (87). In contrast, an increased number of *L. monocytogenes* is found in mouse tissues at the onset of the resting phase as compared with the beginning of the active phase (91). *Salmonella enterica* serovar *Typhimurium* is a foodborne microorganism whose recovery depends on the cell-mediated immune response. In mice, bacterial colonization of the colon is more pronounced during the resting phase than during the active phase. The inflammation induced by *Salmonella* sp. varies similarly. This difference between resting and active time is abolished in clock mutant mice (93). The circadian rhythm pattern of *S. typhimurium* infection reflects circadian rhythm of the innate immune cells. Hence, the ingestion of *S. typhimurium* bacteria by peritoneal macrophages is maximum 16 h after serum-mediated synchronization (111).

In infection due to *Mycobacterium tuberculosis*, it has been shown that cough, a major symptom of tuberculosis, is more frequent during daytime. This is related to highest sputum bacillary load (92). In a mice model, rhythmic melatonin release generated circadian rhythms in granulomatous lesions after inoculation with BCG, an attenuated strain of *M. bovis* used as a vaccine against *M. tuberculosis infection* (112). Moreover, it has been shown that the expression of metalloproteinases by peritoneal macrophages and spleen cells in response to *M. tuberculosis* infection is controlled by the circadian clock in a *Bmal1*-dependent manner (113). Surprisingly, although it is well-established that corticosteroids increase susceptibility to mycobacteria, there is no consensus on the relationship between cortisol circadian rhythms and tuberculosis (114, 115). Fever oscillations during daytime have been reported in Rocky Mountain spotted fever due to *Rickettsia rickettsii*. They consist of an elevated temperature in the evening with a decline early in the morning, while the number of *R. rickettsii* DNA copies is elevated in the blood of patients collected early in the morning, suggesting that rickettsemia has a profile release similar to that of cortisol (116).

Murine models of parasite infection confirm the involvement of circadian rhythm in the evolution of several parasitic infections (98). The moment of infection with the worm *Trichuris muris* affects the kinetics of worm expulsion. Hence, mice infected in the morning expel the parasite early, whereas the parasite persists in mice infected during the night. When the *Bmal1* gene is deleted in antigen-presenting dendritic cells (DCs), the relationship of helminth expulsion and circadian rhythm disappears (99).

The relation between circadian rhythm and the regulation of the viral infection was also reported. Mouse models of infection exhibit daily variations in virus susceptibility: mice inoculated with *Herpes* virus at the beginning of the resting phase (in the daytime) exhibit increased virus load as compared with mice inoculated during the active phase (in the nighttime) (97). Recently it has been shown in a murine model that the severity of influenza An infection depends on the intrinsic pathogenicity of the virus and the uncontrolled inflammatory response. Mice infected before the onset of active phase exhibit higher mortality and morbidity than mice infected before the rest phase. When the *Bmal1* gene is invalidated, the ability to fight the infection is lost through the hyper-inflammation induced by infection at the onset of the active phase (108).

### Clinics: Septic Syndromes

Sepsis is an inflammatory response syndrome that appears after infection, or without documented infection (117). It is the leading cause of death of patients in intensive care units (118) and there is some evidence that circadian rhythm and sepsis are tightly associated (119).

Murine models of endotoxemia that mimic human sepsis provide important information on the mechanisms involved in sepsis and the association between sepsis and circadian rhythm. The administration of lipopolysaccharide (LPS) to mice induces a systemic inflammatory response in which cytokine and chemokine productions are critical. The production of inflammatory molecules including IL-6, IL-12p40, and chemokines, such as CCL2, CCL5, and CXCL1 in response to LPS is higher in mice inoculated early in the active phase than in mice inoculated during the resting phase (120). The endotoxemia in rats is associated with higher levels of inflammatory markers during nighttime than during daytime. In the presence of melatonin, daytime levels of inflammatory markers are increased, underlying the role of melatonin in circadian rhythm coordination in response to LPS (121).

The endotoxemia in young human volunteers is characterized by high levels of IL-10, an anti-inflammatory cytokine, during daytime and high levels of inflammatory cytokines (TNF, IL-1, and IL-6) and cytokine receptors during the nighttime (122). This is consistent with placebo-controlled design studies that show that males receiving LPS in the evening exhibit higher rectal temperature and inflammatory mediator production than those receiving LPS in the morning when cortisol production is the highest (123). There is evidence that LPS response depends on molecular oscillators. Hence, LPS injection induces a lesser production of cytokines in *Clock*^−/−^ bone marrow-derived macrophages than in wild-type cells (93). The engagement of Toll-like receptor (TLR)-9 induces circadian rhythm of TNF and CCL2 with a peak during the active phase; cytokine rhythms are disrupted when the *Per2* gene is invalidated (124). The genes of the molecular clock directly interact promoter regions of TLR genes, thus leading to their circadian rhythm (21).

Clinical studies suggest an association between circadian rhythm disruption and the development of sepsis. In a prospective study, our group assessed the circadian rhythm of cortisol and immune cells in 38 patients at day 2 after severe trauma. The trauma patients who develop an episode of sepsis later in their stay in the intensive care unit were compared to those who did not develop sepsis. The septic patients had higher levels of cortisol than the non-septic patients and delayed acrophases (ie., the peak of production during the period). The acrophases significantly differed between the two groups for lymphocytes, IL-10, and TNF (125). This suggests that the primary insult, represented here by trauma, is associated with disruption of circadian rhythm that affects the host response. In addition, admission to intensive care units is associated with sleep disturbance, sedative infusions and loss of the daily light/dark cycle (126). To counteract these adverse events, the use of melatonin was assessed in three randomized controlled trials (127–129). As the results of these three trials were disappointing, experts made no recommendation on melatonin use in intensive care units (130). Taken together, these findings suggest that circadian disruption is not only associated to sleep dependent of the melatonin, but is multifactorial, and that the initial inflammatory process seems much more critical than the hospitalization in intensive care units.

## Circadian Rhythms and Management of Infected Patients

The investigation of circadian rhythms is well-established in basic research, but its relevance in the medical field remains limited. One of the challenges in the exploration of circadian rhythms in healthy individuals and patients is the high degree of interindividual variability. The circadian rhythm can be evaluated over a period of 24 h with a sampling of at least six points (Table 2). The sampling can be carried out in a longitudinal or transverse manner; longitudinal sampling is useful to obtain the time structure for one individual, whereas transverse or cross-sectional sampling is applied to a group of individuals. The circadian rhythm of individuals is evaluated through measurement of biological variables such as the number of circulating cells, ARN or protein expression of clock molecules or levels of hormones and cytokines. The biological processes associated with the circadian rhythm are also evaluated through physiological variables, such as body temperature (131), blood pressure or wake/sleep cycle. Finally, the circadian pacemaker is directly studied using a bioluminescent system associated with clock molecules. The analysis of biological or physiological variables during the day are based on the Cosinor method developed by Halberg et al. (132). This method enables the measurement of several variables including period, mesor, amplitude and phase of a cycle. These variables require an algorithm-dependent analysis such as Metacycle (133) or, more recently, CircaCompare (134) to estimate and statistically support differences in circadian rhythm (Table 2).

**Table 2:** Circadian rhythm investigation from sampling to data interpretation.

	**Blood/Tissue/Serum**	**Biological process**
Time/Sampling	• Six points minimum over 24 h • Longitudinal sampling (conducted continuously over many cycles, preferably at regular intervals) or • Transverse sampling (sampling of many individuals, once per individual)	
Experiments	Number of circulating immune cell	Temperature, blood pressure, wake/sleep cycle
	ARN expression (clock genes)	
	Protein expression (clock protein)	
	Hormones or cytokines level	
	Bioluminescence monitoring (clock genes, temperature …)	
Analysis	Cosinor and Cosinor fit Parameters: mesor, amplitude, phase, period	
	Statistical tests: Analysis of variance (ANOVA), Metacycle, CircaCompare, Fourier spectral analysis, Singular spectrum analysis (SSA)…	
Interpretation	• Lack of expression • Lack of rhythm • Alteration of the rhythm (modification of one or more parameters) • Increase/decrease in mesor and/or amplitude • Shift/phase change • Lengthening/shortening of the period	

The use of chronobiology in the medical field has been well-documented in pharmacology. Indeed, circadian rhythm is used to determine the better-timed drug delivery, also named chronotherapy (135). This approach associates increased efficacy and reduced toxicity of the drug by the circadian evaluation of its absorption, its metabolism and its elimination (136, 137). The investigation of circadian oscillations in infected patients constitutes a challenge. In patients with sepsis, the measurement of body temperature or blood pressure at a given point instead of circadian manner has been debated (138, 139). We recently reported a circadian dysrhythmia of core body temperature in trauma patients at risk of sepsis, which is associated with increased mortality (131). In another cohort of trauma patients, we showed that the evaluation of the circadian rhythm through measurement of clock gene expression is critical for the identification of a circadian rhythm disruption associated with the occurrence of sepsis (125). The measurement of circadian variations of biological and clinical markers would permit a better stratification of patients, possibly enabling the definition of a chronotype for each infected patient (140).

The vaccination is a relevant example of a situation in which circadian rhythms of host defense and microbes affect the management of patients. Indeed, the antibody response after vaccination depends on the time of administration. In a randomized controlled trial including 276 adults, a significant difference in adaptive response after influenza vaccine administration was observed, with increased antibody production when the vaccine is administered in the morning compared to the afternoon (141). The vaccination of mice with DCs loaded with antigen leads to the expansion of specific CD8^+^ T cells and a better efficiency for a bacterial challenge if the vaccination is done in the middle of the day as compared with other time points. This response is abrogated when the *Bmal1* gene is invalidated in DCs (142) Even if these results are promising, confirmation in other types of vaccination remains required.

It is also likely that the circadian rhythms affect the response to antibiotics. Antibiotic resistance is becoming a major public health problem (143). Interestingly, it has been hypothesized that pathogenic bacteria have an intrinsic circadian rhythm that ensures antibiotic resistance. The resistance to ampicillin, oxacillin, ceftriaxone, meropenem, gentamycin, and ciprofloxacin in clinical strains of *Enterobacteriaceae*, non-fermenting Gram-negative bacilli, and Gram-positive *Staphylococci* has been investigating every 3 h for 24 h. The presence of periods of sensitivity is attested by significant changes in the minimum inhibitory concentrations of antibiotics (144). Similarly, in patients with surgical site infections, variations have been reported in *Staphylococcus aureus* coagulase activity and temporal expression of antibacterial resistance according to the moment of the sampling (145). Further studies will determine the role of circadian rhythm of either the host or the pathogen in this antibiotic resistance process, opening the way for a new strategy of antibiotics administration.

## Conclusion

The analysis of literature has shown that circadian rhythms likely play a role in infectious diseases in terms of susceptibility, clinical expression and outcome. The use of animal models in which clock genes are invalidated has produced a large amount of data supporting the role of circadian pacemakers in the response to microbes. We have also analyzed the mechanisms of the rhythmicity in infectious diseases. It is clear that immune response to pathogens oscillate under the control of peripheral molecular clocks and SCN. The role of corticosteroids has to be considered according to the consequences of its use in patients. Other partners of infectious diseases present rhythms such as microbes and vectors. The identification of molecular rhythms in prokaryotes, particularly so-called “non-circadian” bacteria, will propose a new approach to understand antibiotic resistance. At the interface of immune response and host homeostasis, the rhythms of microbiota provide exciting prospects of understanding metabolic and inflammatory diseases. As circadian rhythms are poised to become biomarkers to assess the outcome of patients with infectious diseases including the risk of complications, new tools for investigating host circadian changes will be required. A clinical chronobiology is necessary to analyze circadian variations at the individual level in infectious diseases. This approach would pave the way for time-based treatment and for administration of molecules known to entrain rhythmicity.

## Author Contributions

AD, BC, ML, SM, and J-LM conceived and wrote the paper. All authors contributed to the article and approved the submitted version.

## Conflict of Interest

The authors declare that the research was conducted in the absence of any commercial or financial relationships that could be construed as a potential conflict of interest.

## Abbreviations

ANOVA: analysis of variance
ARNTL: aryl hydrocarbon receptor nuclear translocator-like
BMAL1: brain and muscle arnt-like protein 1
Ccgs: clock-controlled genes
Ck1: casein kinase 1
CLOCK: circadian locomotor output cycles kaput
CRY: cryptochrome
DBP: d-box binding protein
DCs: dendritic cells
DDT: dichloro-diphenyl-trichloroethane
HC: hepatitis C virus
HSV: herpes simplex virus
Ig: immunoglobulin
IL: interleukin
ILC: innate lymphoid cells
LPS: lipopolysaccharide
miR: microRNA
NFIL3: nuclear factor, interleulin-3 regulated
NK: natural killer
NR1D: nuclear receptor subfamily 1 group D
PER: period
ROR: retinoic acid-related orphan receptor
RORγT: RAR-related orphan receptor gamma
SCN: suprachiasmatic nucleus
SSA: singular spectrum analysis
TLR: toll-like receptor
TNF: tumor necrosis factor.
